# Bis[*S*-octyl 3-(2-methyl­propyl­idene)di­thio­carb­az­ato-κ^2^ *N* ^3^,*S*]nickel(II)

**DOI:** 10.1107/S241431462400186X

**Published:** 2024-03-06

**Authors:** Sultana Shakila Khan, Md. Belayet Hossain Howlader, Md. Chanmiya Sheikh, Ryuta Miyatake, Ennio Zangrando

**Affiliations:** a Department of Chemistry, Rajshahi University, Rajshahi-6205, Bangladesh; b Department of Applied Science, Faculty of Science, Okayama University of Science, Japan; c Center for Environmental Conservation and Research Safety, University of Toyama, 3190 Gofuku, Toyama, 930-8555, Japan; d Department of Chemical and Pharmaceutical Sciences, University of Trieste, Italy; Vienna University of Technology, Austria

**Keywords:** crystal structure, di­thio­carbazato ligand, Ni^II^ complex, *trans* configuration complex, octyl alkyl chain

## Abstract

The central Ni^II^ atom in the title complex, [Ni(C_13_H_25_N_2_S_2_)_2_], is located on an inversion center and adopts a roughly square-planar coordination environment defined by two chelating *N*,*S* donor sets of two symmetry-related ligands in a *trans* configuration. The Ni—N and Ni—S bond lenghts are 1.9193 (14) and 2.1788 (5) Å, respectively, with a chelating N—Ni—S bond angle of 86.05 (4)°. These data are compared with those measured for similar di­thio­carbazato ligands that bear *n*-octyl or *n*-hexyl alkyl chains. Slight differences are observed with respect to the phenyl­ethyl­idene derivative where the ligands are bound *cis* relative to one another.

## Abstract 2

The bis-chelated mononuclear nickel(II) complex shows inversion symmetry, with the di­thio­carbazato ligand in the usual *trans* configuration and the central metal cation in a square-planar coordination environment.

## Graphical abstract

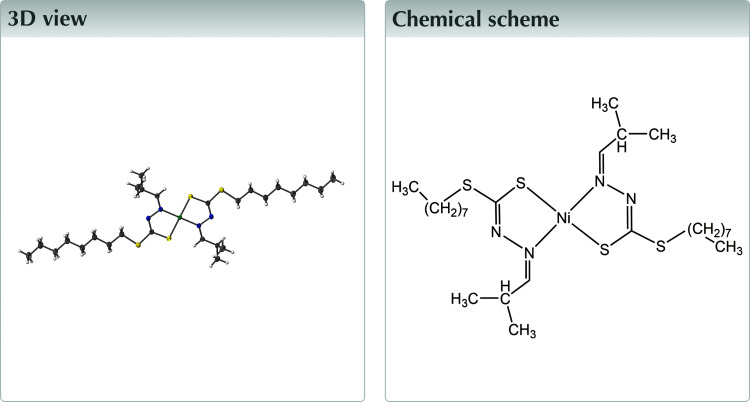

## Structure description

Di­thio­carbazate Schiff base derivatives have emerged as prospective ligands in medicinal chemistry as a result of their various pharmaceutical and biological activities (Gou *et al.*, 2022 ▸; Low *et al.*, 2016 ▸; Malik *et al.*, 2020 ▸). For some years, we have been undertaking a study of *N*,*S*-chelating di­thio­carbazato ligands and their corresponding metal complexes, which were observed to crystallize with ligands both in *trans* and *cis* configurations (Begum *et al.*, 2020 ▸). Considering the above aspects, and in a continuation of our research, we report herein a novel Ni^II^ complex with a di­thio­carbazato Schiff base ligand bearing an octyl alkyl chain.

**Figure 1:**
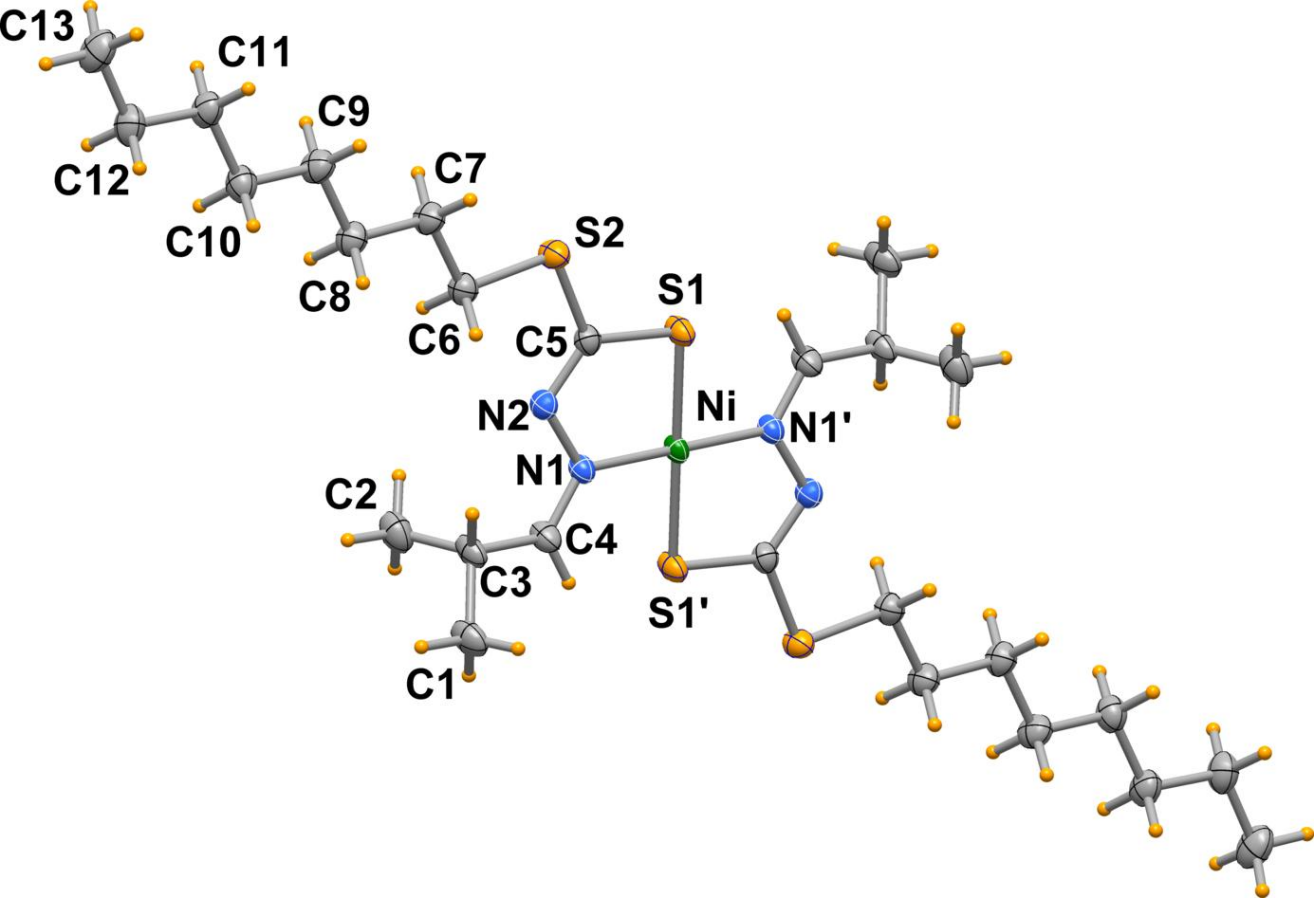
Mol­ecular structure of the title complex [Ni(C_13_H_25_N_2_S_2_)_2_] with displace­ment ellipsoids drawn at the 50% probability level.

In the title complex (Fig. 1 ▸), [Ni(C_13_H_25_N_2_S_2_)_2_], the Ni^II^ atom is located on an inversion center and exhibits a square-planar coordination environment, defined by two negatively charged *N*,*S*-chelating ligands in a *trans* configuration. The Ni—N1 and Ni—S1 bond lengths are 1.9193 (14) and 2.1788 (5) Å, respectively, with a chelating N1—Ni—S1 bond angle of 86.05 (4)°. With the exception of methyl groups C1 and C2, all of the non-H atoms of the title complex are coplanar, with Ni1 (+0.16 Å) and S1 (–0.15 Å) deviating the most from the least-squares plane (r.m.s. deviation of fitted atoms = 0.073 Å). The long alkyl chain is in a staggered conformation with torsion angles along the chain between 178.20 (19) and 179.81 (15)°. The mol­ecular structure is stabilized by an intra­molecular non-conventional C4—H4⋯S1^i^ hydrogen bond with a C4⋯S1^i^ distance of 3.0965 (16) Å and a C4—H4⋯S1^i^ angle of 121° [symmetry code: (i): 1 − *x*, 1 − *y*, 1 − *z*].

A number of Ni^II^ complexes with ligands bearing *n*-octyl or *n*-hexyl alkyl chains have been structurally characterized and a comparison of relevant bond lengths and angles is compiled in Table 1 ▸. The corresponding values reported above are consistent with those measured in bis-chelated Ni^II^ complexes built with similar ligands bearing a meth­oxy­benzyl­idene (Begum *et al.*, 2018 ▸) or thio­phenmethyl­idene (Khan *et al.*, 2023*a* ▸) ligand. On the contrary, in the phenyl­ethyl­idene complex (Khan *et al.*, 2023*b* ▸) the two independent Ni—N bond lengths are 0.02 Å longer with respect to those calculated in the present work, while the Ni—S ones are shorter by *ca* 0.02 Å. It is worth noting that the latter complex exhibits a *cis* configuration of the ligands. A similar trend is also observed in the complex with the *S*-*n*-hexyl 3-(1-phenyl­ethyl­idene)di­thio­carbazate ligand (Begum *et al.*, 2020 ▸). Nickel(II) and copper(II) complexes with di­thio­carbazato ligands have been reported to crystallize in both *cis* and *trans* configurations, although the latter are slightly more frequent (Begum *et al.*, 2020 ▸). However, the chelating *N*,*S* bond angles in these complexes are similar within their standard deviations and fall into the range 85.67 (5)–86.40 (5)°.

**Figure 2:**
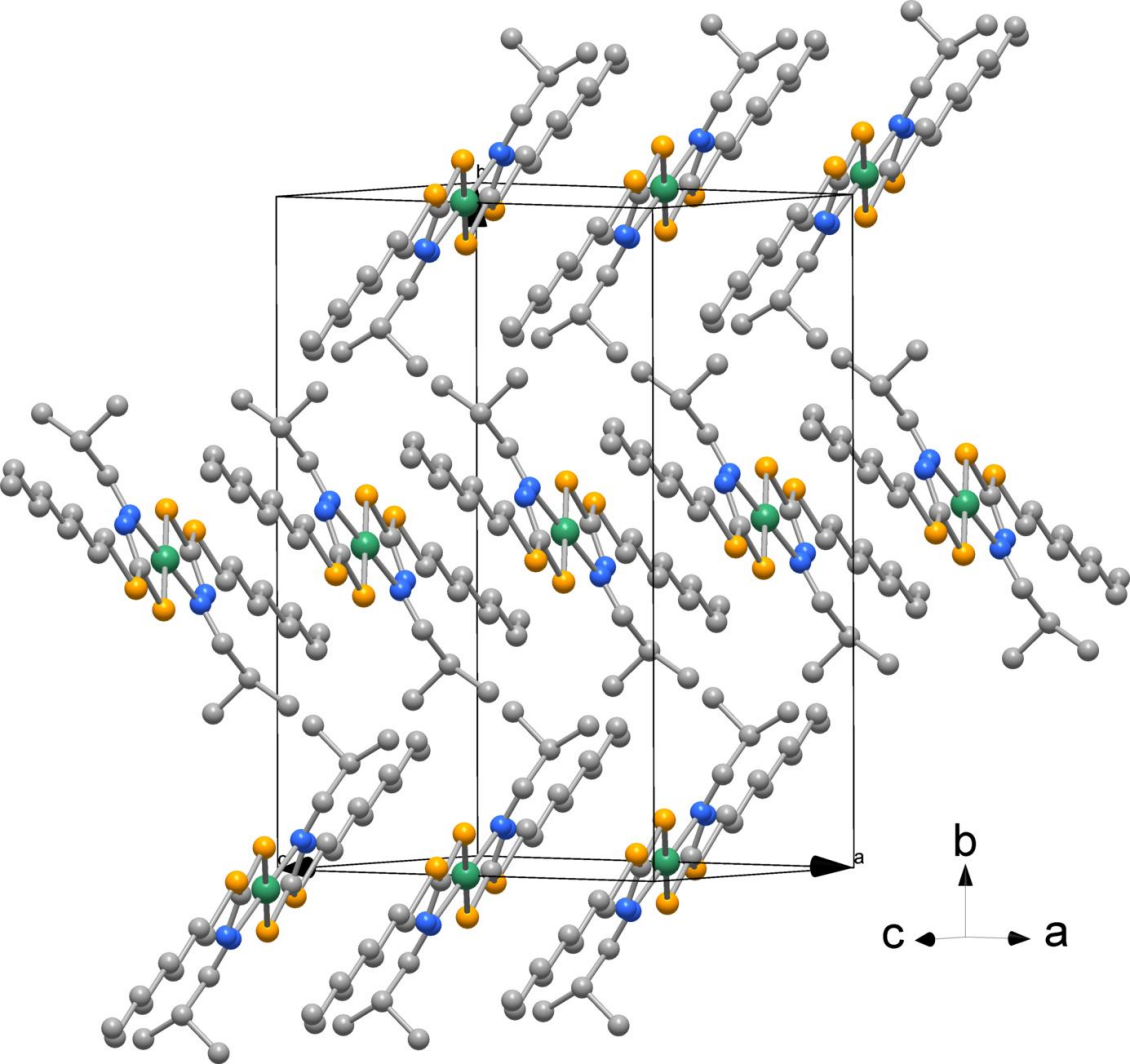
View of the crystal packing of the title complex [Ni(C_13_H_25_N_2_S_2_)_2_] in a view along [102]; H atoms are not shown for clarity.

The packing of the complex is shown in Fig. 2 ▸; the complexes stack with an Ni⋯Ni separation of 7.8973 (2) Å along the *c* axis.

## Synthesis and crystallization

A solution of Ni(CH_3_COO)_2_·4H_2_O (0.124 g, 0.5 mmol) in 10 ml of methanol was added to a solution of *S*-octyl-3-(2-methyl­propyl­idene)di­thio­carbazate (0.274 g, 1.0 mmol) in 30 ml of methanol. The resulting mixture was stirred at room temperature for 5 h. The green precipitate that formed was filtered off, washed with methanol and dried *in vacuo* over anhydrous CaCl_2_. Green single crystals of the title compound suitable for X-ray diffraction were obtained by slow evaporation from a mixture of chloro­form and aceto­nitrile (1:1, *v*/*v*) after 7 d.

Yield: 72%; m. p. 333–334 K. FT–IR (KBr discs, cm^−1^): ν(C—H, alk­yl) 2964, 2922, ν(C=N—N=C) 1634. ^1^H NMR (400 MHz, CDCl_3_, p.p.m.) δ: 6.99 (*d*, 2×1H, C-4, CH=N), 3.29 (*m*, 2×1H, C-3), 2.98 (*t*, 2×2H, C-6, –SCH_2_), 1.65 (*m*, 2×2H, C-7), 1.38–1.12 (*m*, 2×10H, C-8, 9, 10, 11, 12), 1.00 (*d*, 2×6H, C-1, 2, CH_3_), 0.87 (*t*, 2×3H, C-13, CH_3_). HRMS (FAB) Calculated for C_26_H_50_N_4_NiS_4_ [*M* + H]^+^: 605.23494, found [*M* + H]^+^: 605.23445.

## Refinement

Crystal data, data collection and structure refinement details are summarized in Table 2 ▸.

## Supplementary Material

Crystal structure: contains datablock(s) global, I. DOI: 10.1107/S241431462400186X/wm4208sup1.cif

Structure factors: contains datablock(s) I. DOI: 10.1107/S241431462400186X/wm4208Isup2.hkl

CCDC reference: 2335121

Additional supporting information:  crystallographic information; 3D view; checkCIF report

## Figures and Tables

**Table 1:** Comparative geometrical parameters (Å, °) for bis-chelated Ni complexes with di­thio­carbazato ligands bearing an *S*-oct­yl/*S*-hexyl (*n*) alkyl chain

Complex	CSD Refcode	*n*	Ni—N	Ni—S	N—Ni—S
This work	–	8	1.9193 (14)	2.1788 (5)	86.05 (4)
1	BIQTIH	8	1.9310 (19)	2.1796 (6)	85.67 (5)
2	MIMTIG	8	1.9168 (19)	2.1735 (7)	85.88 (6)
3, ligand 1	QIVYUT	8	1.9318 (16)	2.1506 (6)	86.26 (5)
3, ligand 2	QIVYUT	8	1.9392 (16)	2.1573 (6)	86.40 (5)
4	LUBYAK	6	1.933 (3)	2.1775 (10)	86.04 (9)
5, ligand 1	JUYCAJ	6	1.9112 (12)	2.1785 (4)	85.74 (3)
5, ligand 2	JUYCAJ	6	1.9177 (12)	2.1812 (4)	86.03 (4)
6	WEGKEB	6	1.915 (2)	2.1788 (8)	85.58 (8)
7	TILVUJ	6	1.9295 (10)	2.1600 (4)	85.68 (3)

Notes: Complex **1** (Begum *et al.*, 2018 ▸); **2** (Kahn *et al.*, 2023*a* ▸); **3** (Kahn *et al.*, 2023*b* ▸); **4** (Howlader *et al.*, 2015 ▸); **5** (Begum *et al.*, 2016 ▸); **6** (Begum *et al.*, 2017 ▸); **7** (Begum *et al.*, 2020 ▸). Complexes **3** and **7** show a *cis* configuration of ligands.

**Table 2:** Experimental details

Crystal data	
Chemical formula	[Ni(C_13_H_25_N_2_S_2_)_2_]
*M*_r_	605.65
Crystal system, space group	Monoclinic, *P*2_1_/*c*
Temperature (K)	173
*a*, *b*, *c* (Å)	11.5962 (4), 18.4606 (5), 7.8973 (2)
β (°)	106.532 (7)
*V* (Å^3^)	1620.71 (10)
*Z*	2
Radiation type	Mo *K*α
μ (mm^−1^)	0.88
Crystal size (mm)	0.18 × 0.06 × 0.02

Data collection	
Diffractometer	Rigaku R-AXIS RAPID
Absorption correction	Multi-scan (*ABSCOR*; Rigaku, 1995 ▸)
*T*_min_, *T*_max_	0.781, 0.983
No. of measured, independent and observed [*I* > 2σ(*I*)] reflections	15550, 3702, 2920
*R*_int_	0.049
(sin θ/λ)_max_ (Å^−1^)	0.649

Refinement	
*R*[*F* ^2^ > 2σ(*F* ^2^)], *wR*(*F* ^2^), *S*	0.036, 0.072, 1.04
No. of reflections	3702
No. of parameters	163
H-atom treatment	H-atom parameters constrained
Δρ_max_, Δρ_min_ (e Å^−3^)	0.37, −0.21

Computer programs: *RAPID-AUTO* (Rigaku, 2018 ▸), *SHELXT* (Sheldrick, 2015*a* ▸), *SHELXL* (Sheldrick, 2015*b* ▸), *DIAMOND* (Brandenburg, 1999 ▸) and *WinGX publication routines* (Farrugia, 2012 ▸).

## full crystallographic data

### Crystal data

**Table:**

[Ni(C_13_H_25_N_2_S_2_)_2_]	*F*(000) = 652
*M**_r_* = 605.65	*D*_x_ = 1.241 Mg m^−^^3^
Monoclinic, *P*2_1_/*c*	Mo *K*α radiation, λ = 0.71075 Å
*a* = 11.5962 (4) Å	Cell parameters from 11425 reflections
*b* = 18.4606 (5) Å	θ = 1.8–27.5°
*c* = 7.8973 (2) Å	µ = 0.88 mm^−^^1^
β = 106.532 (7)°	*T* = 173 K
*V* = 1620.71 (10) Å^3^	Platelet, green
*Z* = 2	0.18 × 0.06 × 0.02 mm

### Data collection

**Table:**

Rigaku R-AXIS RAPID diffractometer	2920 reflections with *I* > 2σ(*I*)
Detector resolution: 10.000 pixels mm^-1^	*R*_int_ = 0.049
ω scans	θ_max_ = 27.5°, θ_min_ = 2.2°
Absorption correction: multi-scan (ABSCOR; Rigaku, 1995)	*h* = −15→15
*T*_min_ = 0.781, *T*_max_ = 0.983	*k* = −23→23
15550 measured reflections	*l* = −10→9
3702 independent reflections	

### Refinement

**Table:**

Refinement on *F*^2^	0 restraints
Least-squares matrix: full	Hydrogen site location: inferred from neighbouring sites
*R*[*F*^2^ > 2σ(*F*^2^)] = 0.036	H-atom parameters constrained
*wR*(*F*^2^) = 0.072	*w* = 1/[σ^2^(*F*_o_^2^) + (0.0305*P*)^2^ + 0.4396*P*] where *P* = (*F*_o_^2^ + 2*F*_c_^2^)/3
*S* = 1.04	(Δ/σ)_max_ = 0.001
3702 reflections	Δρ_max_ = 0.37 e Å^−^^3^
163 parameters	Δρ_min_ = −0.21 e Å^−^^3^

### Special details

**Table:**

Geometry. All esds (except the esd in the dihedral angle between two l.s. planes) are estimated using the full covariance matrix. The cell esds are taken into account individually in the estimation of esds in distances, angles and torsion angles; correlations between esds in cell parameters are only used when they are defined by crystal symmetry. An approximate (isotropic) treatment of cell esds is used for estimating esds involving l.s. planes.

### Fractional atomic coordinates and isotropic or equivalent isotropic displacement parameters (Å^2^)

**Table:**

	*x*	*y*	*z*	*U*_iso_*/*U*_eq_	
Ni1	0.500000	0.500000	0.500000	0.02167 (9)	
S1	0.60829 (5)	0.43196 (2)	0.71132 (6)	0.02979 (12)	
S2	0.72948 (5)	0.47341 (3)	1.07771 (6)	0.02978 (12)	
N1	0.50276 (14)	0.56970 (8)	0.68145 (18)	0.0234 (3)	
N2	0.58197 (14)	0.56067 (8)	0.85404 (18)	0.0256 (3)	
C1	0.3208 (2)	0.71961 (11)	0.7769 (3)	0.0404 (5)	
H1A	0.324068	0.754490	0.871744	0.049*	
H1B	0.258003	0.683641	0.773519	0.049*	
H1C	0.302375	0.745114	0.663553	0.049*	
C2	0.5430 (2)	0.73600 (11)	0.8188 (3)	0.0428 (5)	
H2A	0.526392	0.761787	0.705932	0.051*	
H2B	0.619737	0.710216	0.841194	0.051*	
H2C	0.547434	0.770833	0.914072	0.051*	
C3	0.44208 (19)	0.68158 (10)	0.8112 (2)	0.0313 (5)	
H3	0.459840	0.655777	0.927134	0.038*	
C4	0.43794 (19)	0.62718 (10)	0.6691 (2)	0.0296 (4)	
H4	0.381339	0.636002	0.557753	0.035*	
C5	0.63166 (16)	0.49751 (10)	0.8740 (2)	0.0236 (4)	
C6	0.71997 (18)	0.54982 (10)	1.2175 (2)	0.0284 (4)	
H6A	0.746134	0.594707	1.170536	0.034*	
H6B	0.636015	0.556435	1.221253	0.034*	
C7	0.80161 (19)	0.53402 (11)	1.4016 (2)	0.0318 (4)	
H7A	0.774890	0.488545	1.445197	0.038*	
H7B	0.884609	0.526391	1.394499	0.038*	
C8	0.80273 (18)	0.59419 (11)	1.5335 (2)	0.0318 (4)	
H8A	0.720371	0.600857	1.544146	0.038*	
H8B	0.827396	0.640071	1.488945	0.038*	
C9	0.88802 (19)	0.57782 (12)	1.7150 (2)	0.0348 (5)	
H9A	0.864291	0.531126	1.756945	0.042*	
H9B	0.970303	0.571941	1.703476	0.042*	
C10	0.8907 (2)	0.63547 (11)	1.8535 (2)	0.0351 (5)	
H10A	0.809207	0.640256	1.868614	0.042*	
H10B	0.912217	0.682544	1.810629	0.042*	
C11	0.97951 (19)	0.61888 (12)	2.0320 (2)	0.0349 (5)	
H11A	0.956543	0.572383	2.075870	0.042*	
H11B	1.060468	0.612554	2.015773	0.042*	
C12	0.9864 (2)	0.67654 (12)	2.1710 (3)	0.0391 (5)	
H12A	0.905293	0.683647	2.185985	0.047*	
H12B	1.011361	0.722853	2.128801	0.047*	
C13	1.0734 (2)	0.65819 (14)	2.3487 (3)	0.0470 (6)	
H13A	1.153265	0.648814	2.334234	0.056*	
H13B	1.045122	0.614956	2.396874	0.056*	
H13C	1.078151	0.698927	2.429976	0.056*	

### Atomic displacement parameters (Å^2^)

**Table:**

	*U*^11^	*U*^22^	*U*^33^	*U*^12^	*U*^13^	*U*^23^
Ni1	0.02940 (18)	0.01765 (16)	0.01852 (16)	0.00189 (14)	0.00771 (13)	−0.00255 (13)
S1	0.0423 (3)	0.0221 (2)	0.0226 (2)	0.0069 (2)	0.0055 (2)	−0.00327 (19)
S2	0.0348 (3)	0.0295 (2)	0.0225 (2)	0.0061 (2)	0.0041 (2)	−0.0013 (2)
N1	0.0317 (9)	0.0195 (7)	0.0187 (7)	0.0016 (6)	0.0066 (6)	−0.0007 (6)
N2	0.0314 (9)	0.0245 (8)	0.0193 (7)	0.0011 (6)	0.0050 (6)	−0.0028 (6)
C1	0.0578 (15)	0.0326 (11)	0.0314 (11)	0.0130 (10)	0.0134 (10)	−0.0055 (9)
C2	0.0600 (15)	0.0315 (11)	0.0382 (11)	0.0013 (10)	0.0159 (11)	−0.0078 (9)
C3	0.0490 (13)	0.0223 (9)	0.0220 (9)	0.0080 (9)	0.0091 (9)	−0.0031 (8)
C4	0.0434 (12)	0.0243 (9)	0.0203 (9)	0.0065 (8)	0.0080 (8)	0.0005 (8)
C5	0.0276 (9)	0.0232 (8)	0.0209 (8)	−0.0011 (8)	0.0082 (7)	−0.0016 (8)
C6	0.0313 (11)	0.0297 (10)	0.0244 (9)	0.0006 (8)	0.0083 (8)	−0.0027 (8)
C7	0.0351 (11)	0.0349 (11)	0.0234 (9)	0.0026 (9)	0.0051 (8)	−0.0035 (9)
C8	0.0354 (11)	0.0355 (10)	0.0236 (9)	−0.0011 (9)	0.0069 (8)	−0.0033 (9)
C9	0.0363 (11)	0.0414 (12)	0.0245 (9)	0.0019 (9)	0.0053 (8)	−0.0037 (9)
C10	0.0434 (13)	0.0330 (11)	0.0278 (10)	−0.0023 (9)	0.0082 (9)	−0.0041 (9)
C11	0.0360 (12)	0.0395 (11)	0.0281 (10)	−0.0012 (9)	0.0073 (9)	−0.0083 (9)
C12	0.0469 (13)	0.0388 (12)	0.0314 (10)	−0.0079 (10)	0.0110 (9)	−0.0087 (9)
C13	0.0447 (14)	0.0614 (15)	0.0323 (11)	−0.0070 (11)	0.0069 (10)	−0.0129 (11)

### Geometric parameters (Å, º)

**Table:**

Ni1—N1	1.9193 (14)	C6—H6B	0.9900
Ni1—N1^i^	1.9193 (14)	C7—C8	1.520 (3)
Ni1—S1^i^	2.1788 (5)	C7—H7A	0.9900
Ni1—S1	2.1788 (5)	C7—H7B	0.9900
S1—C5	1.7295 (18)	C8—C9	1.522 (3)
S2—C5	1.7416 (18)	C8—H8A	0.9900
S2—C6	1.8138 (19)	C8—H8B	0.9900
N1—C4	1.288 (2)	C9—C10	1.520 (3)
N1—N2	1.420 (2)	C9—H9A	0.9900
N2—C5	1.290 (2)	C9—H9B	0.9900
C1—C3	1.526 (3)	C10—C11	1.521 (3)
C1—H1A	0.9800	C10—H10A	0.9900
C1—H1B	0.9800	C10—H10B	0.9900
C1—H1C	0.9800	C11—C12	1.514 (3)
C2—C3	1.530 (3)	C11—H11A	0.9900
C2—H2A	0.9800	C11—H11B	0.9900
C2—H2B	0.9800	C12—C13	1.516 (3)
C2—H2C	0.9800	C12—H12A	0.9900
C3—C4	1.496 (2)	C12—H12B	0.9900
C3—H3	1.0000	C13—H13A	0.9800
C4—H4	0.9500	C13—H13B	0.9800
C6—C7	1.521 (2)	C13—H13C	0.9800
C6—H6A	0.9900		
			
N1—Ni1—N1^i^	180.0	C8—C7—C6	113.37 (16)
N1—Ni1—S1^i^	93.95 (4)	C8—C7—H7A	108.9
N1^i^—Ni1—S1^i^	86.05 (4)	C6—C7—H7A	108.9
N1—Ni1—S1	86.05 (4)	C8—C7—H7B	108.9
N1^i^—Ni1—S1	93.95 (4)	C6—C7—H7B	108.9
S1^i^—Ni1—S1	180.00 (2)	H7A—C7—H7B	107.7
C5—S1—Ni1	95.74 (6)	C7—C8—C9	112.13 (16)
C5—S2—C6	103.11 (8)	C7—C8—H8A	109.2
C4—N1—N2	111.92 (14)	C9—C8—H8A	109.2
C4—N1—Ni1	127.63 (13)	C7—C8—H8B	109.2
N2—N1—Ni1	120.45 (11)	C9—C8—H8B	109.2
C5—N2—N1	111.59 (14)	H8A—C8—H8B	107.9
C3—C1—H1A	109.5	C10—C9—C8	114.43 (17)
C3—C1—H1B	109.5	C10—C9—H9A	108.7
H1A—C1—H1B	109.5	C8—C9—H9A	108.7
C3—C1—H1C	109.5	C10—C9—H9B	108.7
H1A—C1—H1C	109.5	C8—C9—H9B	108.7
H1B—C1—H1C	109.5	H9A—C9—H9B	107.6
C3—C2—H2A	109.5	C9—C10—C11	113.32 (17)
C3—C2—H2B	109.5	C9—C10—H10A	108.9
H2A—C2—H2B	109.5	C11—C10—H10A	108.9
C3—C2—H2C	109.5	C9—C10—H10B	108.9
H2A—C2—H2C	109.5	C11—C10—H10B	108.9
H2B—C2—H2C	109.5	H10A—C10—H10B	107.7
C4—C3—C1	110.15 (16)	C12—C11—C10	114.35 (17)
C4—C3—C2	109.32 (17)	C12—C11—H11A	108.7
C1—C3—C2	111.16 (17)	C10—C11—H11A	108.7
C4—C3—H3	108.7	C12—C11—H11B	108.7
C1—C3—H3	108.7	C10—C11—H11B	108.7
C2—C3—H3	108.7	H11A—C11—H11B	107.6
N1—C4—C3	127.07 (17)	C11—C12—C13	113.45 (18)
N1—C4—H4	116.5	C11—C12—H12A	108.9
C3—C4—H4	116.5	C13—C12—H12A	108.9
N2—C5—S1	124.84 (14)	C11—C12—H12B	108.9
N2—C5—S2	119.66 (13)	C13—C12—H12B	108.9
S1—C5—S2	115.50 (10)	H12A—C12—H12B	107.7
C7—C6—S2	107.68 (13)	C12—C13—H13A	109.5
C7—C6—H6A	110.2	C12—C13—H13B	109.5
S2—C6—H6A	110.2	H13A—C13—H13B	109.5
C7—C6—H6B	110.2	C12—C13—H13C	109.5
S2—C6—H6B	110.2	H13A—C13—H13C	109.5
H6A—C6—H6B	108.5	H13B—C13—H13C	109.5
			
C4—N1—N2—C5	169.13 (17)	C6—S2—C5—N2	5.12 (18)
Ni1—N1—N2—C5	−10.2 (2)	C6—S2—C5—S1	−174.45 (11)
N2—N1—C4—C3	1.5 (3)	C5—S2—C6—C7	179.27 (14)
Ni1—N1—C4—C3	−179.19 (15)	S2—C6—C7—C8	179.81 (15)
C1—C3—C4—N1	−154.1 (2)	C6—C7—C8—C9	−178.30 (18)
C2—C3—C4—N1	83.5 (2)	C7—C8—C9—C10	−178.72 (18)
N1—N2—C5—S1	1.2 (2)	C8—C9—C10—C11	−178.20 (19)
N1—N2—C5—S2	−178.29 (12)	C9—C10—C11—C12	178.39 (19)
Ni1—S1—C5—N2	6.18 (17)	C10—C11—C12—C13	178.8 (2)
Ni1—S1—C5—S2	−174.28 (9)		

Symmetry code: (i) −*x*+1, −*y*+1, −*z*+1.

## References

[bb1] Begum, K., Begum, S., Sheikh, C., Miyatake, R. & Zangrando, E. (2020). *Acta Cryst.* E**76**, 692–696. 10.1107/S205698902000506X PMC7199265 32431934

[bb2] Begum, K., Zangrando, E., Begum, M. S., Sheikh, C. & Miyatake, R. (2018). *IUCrData*, **3**, x181684.

[bb3] Begum, M. S., Zangrando, E., Howlader, M. B. H., Sheikh, M. C., Miyatake, R., Hossain, M. M., Alam, M. M. & Hasnat, M. A. (2016). *Polyhedron*, **105**, 56–61.

[bb4] Begum, M. S., Zangrando, E., Sheikh, M. C., Miyatake, R., Howlader, M. B. H., Rahman, M. N. & Ghosh, A. (2017). *Transit. Met. Chem.* **42**, 553–563.

[bb5] Brandenburg, K. (1999). *DIAMOND.* Crystal Impact GbR, Bonn, Germany.

[bb6] Farrugia, L. J. (2012). *J. Appl. Cryst.* **45**, 849–854.

[bb7] Gou, Y., Jia, X., Hou, L. X., Deng, J. G., Huang, G. J., Jiang, H. W. & Yang, F. (2022). *J. Med. Chem.* **65**, 6677–6689. 10.1021/acs.jmedchem.1c02186 35446587

[bb8] Howlader, M. B. H., Begum, M. S., Sheikh, M. C., Miyatake, R. & Zangrando, E. (2015). *Acta Cryst.* E**71**, m26–m27. 10.1107/S2056989015000328 PMC4384570 25878838

[bb9] Khan, S. S., Howlader, M. B. H., Sheikh, M. C., Miyatake, R. & Zangrando, E. (2023*a*). *Acta Cryst.* E**79**, 714–717. 10.1107/S2056989023005935 PMC10439407 37601399

[bb10] Khan, S. S., Howlader, M. B. H., Sheikh, M. C., Miyatake, R., Zangrando, E. & Ansary, M. R. H. (2023*b*). *Acta Cryst.* E**79**, 1137–1141. 10.1107/S2056989023009726 PMC10833409 38313117

[bb11] Low, M. L., Maigre, L., Tahir, M. I., Tiekink, E. R., Dorlet, P., Guillot, R., Ravoof, T. B., Rosli, R., Pagès, J. M., Policar, C., Delsuc, N. & Crouse, K. A. (2016). *Eur. J. Med. Chem.* **120**, 1–12. 10.1016/j.ejmech.2016.04.027 27183379

[bb12] Malik, M. A., Lone, S. A., Wani, M. Y., Talukdar, M. I. A., Dar, O. A., Ahmad, A. & Hashmi, A. A. (2020). *Bioorg. Chem.* **98**, 103771. 10.1016/j.bioorg.2020.103771 32224354

[bb13] Rigaku (1995). *ABSCOR*. Rigaku Corporation, Tokyo, Japan.

[bb14] Rigaku (2018). *RAPID AUTO*. Rigaku Corporation, Tokyo, Japan.

[bb15] Sheldrick, G. M. (2015*a*). *Acta Cryst.* A**71**, 3–8.

[bb16] Sheldrick, G. M. (2015*b*). *Acta Cryst.* C**71**, 3–8.

